# Upregulation of miR-96 Enhances Cellular Proliferation of Prostate Cancer Cells through FOXO1

**DOI:** 10.1371/journal.pone.0072400

**Published:** 2013-08-12

**Authors:** Benedikta S. Haflidadóttir, Olivia Larne, Myriam Martin, Margareta Persson, Anders Edsjö, Anders Bjartell, Yvonne Ceder

**Affiliations:** 1 Department of Laboratory Medicine, Division of Clinical Chemistry, Lund University, Malmö, Sweden; 2 Department of Laboratory Medicine, Division of Medical Protein Chemistry, Lund University, Malmö, Sweden; 3 Department of Laboratory Medicine, Center for Molecular Pathology, Lund University, Malmö, Sweden; 4 Department of Clinical Sciences, Division of Urological Cancers, Lund University, Malmö, Sweden; Queensland University of Technology, Australia

## Abstract

Aberrant expression of miR-96 in prostate cancer has previously been reported. However, the role and mechanism of action of miR-96 in prostate cancer has not been determined. In this study, the diagnostic and prognostic properties of miR-96 expression levels were investigated by qRT-PCR in two well documented prostate cancer cohorts. The miR-96 expression was found to be significantly higher in prostate cancer patients and correlate with WHO grade, and decreased overall survival time; patients with low levels of miR-96 lived 1.5 years longer than patients with high miR-96 levels. The therapeutic potential was further investigated in vitro, showing that ectopic levels of miR-96 enhances growth and cellular proliferation in prostate cancer cells, implying that miR-96 has oncogenic properties in this setting. We demonstrate that miR-96 expression decreases the transcript and protein levels of FOXO1 by binding to one of two predicted binding sites in the FOXO1 3'UTR sequence. Blocking this binding site completely inhibited the growth enhancement conveyed by miR-96. This finding was corroborated in a large external prostate cancer patient cohort where miR-96 expression inversely correlated to FOXO1 expression. Taken together these findings indicate that miR-96 plays a key role in prostate cancer cellular proliferation and can enhance prostate cancer progression. This knowledge might be utilized for the development of novel therapeutic tools for prostate cancer.

## Introduction

Prostate cancer (PCa) is the most common cancer in European and North American men and one of the main causes of cancer related deaths [1]. While confined to the prostate gland, the cancer is curable by either prostatectomy or radiation therapy [2]. As the tumor progresses, it develops the abilities to invade surrounding tissue, induce angiogenesis, and metastasize. Androgen deprivation therapy, either chemical or surgical castration, is the gold standard treatment for advanced PCa. This treatment option results in significant clinical regression in almost all patients [3,4]. However, the majority of the tumors become castration resistant and resumes growth within 12-18 months and for recurrent tumors only palliative therapies are available. To survive and resume growth in an androgen depleted surrounding, the cells must either adapt the androgen receptor (AR) pathway or induce alternative survival and growth pathways. Mechanisms underlying adaptation of the AR can be increased expression of the AR, increased local production of androgens, hypersensitivity or constitutively active truncated forms of the AR, promiscuity, and/or ligand independent activation through kinase cross-talk. In PCa deregulated microRNA (miRNA) expression has been reported [5–7] and miRNAs are believed to contribute to the tumor progression through their involvement in cell proliferation, apoptosis, invasion, metastasis and castration resistance onset [reviewed in 8–10]. We and others have previously shown that miR-96 levels are upregulated in PCa [5,7,11] and that it is also highly expressed in several other cancer types, including lymphoma, liver, breast, ovarian, lung, colon, testicular and colorectal cancer [5,12]. miR-96 has been suggested to act as an oncomiR regulating proliferation and DNA repair [13], but also as a tumor suppressor inducing apoptosis in pancreatic cells [14]. In breast cancer, miR-96 promotes cell proliferation through targeting the tumor suppressor gene Forkhead box O transcription factor, FOXO3a, and the cyclin-dependent kinase inhibitors p27^Kip1^ and p21^Cip1^ [15]. miR-96 has also been shown to target FOXO1 in endometrial [16], breast [17], hepatocellular cancer cells [18] and Hodgkin lymphoma [19]. Forkhead box O proteins FOXO1, FOXO3a, FOXO4, and FOXO6 are transcription factors involved in biological processes such as DNA damage repair [20], cell cycle [21,22] and apoptosis [21,23]. The FOXO1 tumor suppressor is located at 13q41, an area often deleted in PCa and other cancers, and both nuclear FOXO1 and transcript levels have been shown to be decreased in PCa [24,25]. Phosphatase and tensin homolog (PTEN) is often lost in prostate cancer [26,27] which would also lead to loss or decreased function of downstream effectors such as FOXO1 [21]. FOXO1 has been shown to enhance apoptosis [17,21] and decrease proliferation [17,21]. In PCa cells specifically, FOXO1 induces apoptosis and cell cycle arrest [21,28], and has also been shown to be a part of a regulatory feedback loop with the AR in PCa. FOXO1 represses both the androgen-dependent and androgen-independent activity of AR [24,29,30], and AR inhibits the DNA binding activity of FOXO1 by forming a protein–protein complex with FOXO1, which renders FOXO1 unable to induce apoptosis and cell cycle arrest [30].

Hence, we hypothesized that in PCa, miR-96 act as an oncomiR, affecting tumor progression. In this study, the prognostic properties of miR-96 were analyzed in two cohorts of PCa patients and the expression correlated to clinical parameters. The effect of miR-96 on cell growth and proliferation was assessed *in vitro* and FOXO1 was identified as a direct target of miR-96 in PCa cells.

## Materials and Methods

### Ethics statement

Ethical approval for all samples described has been obtained from "Regionala etikprövningsnämnden i Lund" (the local Ethical Review Board in Lund, Sweden), approval #: LU909-03 and the personal data anonymized. The ethics committee waived the need for written consent and on their suggestion information about the research containing instructions of opt-out the procedure was published in all major local newspapers. We adhere to the declaration of Helsinki and the Data Protection Directive.

### Patient samples

Cohort 1, previously described [31,32] and in table S1, was used to analyse miR-96 expression. It consists of tissue samples collected from transurethral resection of the prostate (TURPs), collected 1990-1999 in Malmö, Sweden. Briefly, the material was fixed in 4% buffered paraformaldehyde and paraffin embedded. The samples were graded according to the WHO standard and the diagnosis was based on histopathological diagnosis in randomly selected cases with evidence of prostate adenocarcinoma in 50 patients and benign prostatic hyperplasia (BPH) (i.e., no evidence of PCa) in another 25 men. The presence of PCa and assessment of the amount (%) of cancer cells was done using sections adjacent to those used for miRNA analyses [31]. One (1/50) cancer sample was not found to contain PCa in the adjacent section and was excluded from the final data set. The age range at time of TURP was 63–89, with a mean of 76 years for the men diagnosed with cancer, and 56–86, with a mean of 71 years for the men with BPH. Cohort 2 consists of 93 formalin fixed paraffin embedded (FFPEs) tissues obtained from radical prostatectomies, graded according to WHO and Gleason. The samples were collected at Malmö Hospital 1999–2002 and are described in table S2. The age range at time of prostatectomy was 48-73 with a mean of 62 years. Appropriate ethical approvals have been obtained from the Ethics Committee, Lund University and we have adhered to the Helsinki Declaration.

### Cell culture and transfection

PCa cell lines 22Rv1, LNCaP clone FGC, DU145 and PC3 were obtained from American Type Culture Collection and VCaP and PNT2 cell lines were obtained from European Collection of Cell Culture. The cells were cultured according to the supplier’s recommendations. Cells were transiently transfected with miRIDIAN microRNA Mimic (C-300514-07, 80nM probe, Thermo Fisher Scientific Inc., Wilmington, DE) and in parallel; cells were transfected with miRIDIAN microRNA Mimic Negative Control (CN-001000-01-05). To inhibit endogenous miR-96, cells were transfected with miRCury LNA inhibitors (100nM probe, Exiqon A/S, Vedbaek, Denmark), miR-96 inhibitor (Cat. no. 410467-00) and in parallel with Negative Control A (Cat. no. 199004-00). Cells were transfected using Oligofectamin reagent (Invitrogen, Carlsbad, CA).

### Isolation of RNA

The RNA isolation from the PCa and BPH tissue samples in cohort 1 was previously described [31]. Briefly, RNA was extracted from 20µm sections of 75 formalin fixed paraffin embedded (FFPEs) prostate tissue samples. Small RNAs were extracted with a slightly modified protocol of mirVanaTM miRNA Isolation Kit (Ambion); the samples were deparaffinised by xylene treatment and digested by protease before the organic extraction. After washing the filter containing the small RNA, the samples were DNase treated (RecoverAll, Ambion), and washed again. In cohort 2, total RNA was extracted from prostate tissue cores (1-4mm). Total RNA was extracted according to a modified protocol of *mir*Vana™ miRNA Isolation Kit (Ambion) as described in Hagman et al. [31]. All RNA concentrations were measured using a NanoDrop (ND-1000, Spectrophotometer, Thermo Fisher Scientific Inc.). The RNA extraction from 27 human tissues of various origin has been described previously [33]. From the cell lines, total RNA was isolated using Trizol reagent according to the manufacturer’s instructions (Invitrogen), and treated with DNase (Promega Biosciences, San Luis Obispo, CA). The RNA concentration was measured using a NanoDrop. For external validation of the correlation between miR-96 and FOXO1 transcript levels we analyzed an external microarray data set from Taylor et al. constituting 110 prostate cancer tissue samples and 28 non-malignant adjacent benign prostate tissue samples [34] (GEO accession number GSE21036).

### Reverse transcription reaction and qRT-PCR

The miRNA levels were quantified by TaqMan Micro-RNA Assays protocol (Applied Biosystems, Foster City, CA) according to the manufacturer’s instructions with minor changes. Briefly, 5 or 10ng small RNAs were reversely transcribed with miR-96 specific primers (Assay no. 000186). The RT product was amplified in 10µl reactions by qRT-PCR in 384-well plates on a 7900 HT Fast Real-Time PCR System (Applied Biosystems). The samples were run in quadruplicates, and quantification was performed by the comparative Delta Ct method. Log_2_-transformed values were normalized by dividing with the geometric mean of the 3 housekeeping genes RNU48, RNU66 and U47 in the study of patient samples. In the study of miR-96 expression in tissues of different human origin and PCa cell lines the geometric mean of RNU48, RNU66, RNU24 and RNU44 was used. The expression of FOXO1 (primer: Hs01054576m1) in cell lines and after miR-96 overexpression in 22Rv1 cells, the mean of GAPDH (Hs02758991_g1), and PGK1 (Hs9999906) was used as control. These housekeeping genes also served as control for the RNA integrity. Along with the reverse transcription and the qRT-PCR, a no enzyme negative control and a no template control were run to exclude PCR contamination and genomic DNA.

### Cell number and cell growth

Cell number was counted in triplicates of samples transfected with miR-96 mimic compared to a negative control at four time points, 2, 3, 4 and 5 days after transfection. Cells were trypzinised and counted on a Bürkner chamber. Sulforhodamine B (SRB) assay was used to measure cell growth indirectly by staining the total protein content of cells transfected with miR-96 mimics compared to cells transfected with the negative control. Cells were fixed in ice-cold 10% Trichloroacetic acid and stained with 0.4% SRB (S9012-5G from Sigma-Aldrich Co, St. Louis, MO) in 1% acetic acid for 15 min. Unbound SRB was washed off with 1% acetic acid. Bound SRB was dissolved in 10mM Tris base and the absorbance was read at 490nm using ELx808 IU Ultra Microplate Reader (Biotek Instruments, Inc, Winooski, VT). Cells transfected with miR-96 mimics were harvested 72-120 h after transfection, DU145 cells were harvested 72 h after transfection, PC3 cells 96 h and 22Rv1 cells 120 h after transfection.

### Cell proliferation

Cells transfected with miR-96 or the negative control (in triplicate) were incubated with 5-bromo-2 deoxyuridine (BrdU, GE healthcare, Wauwatausa, WI) at a dilution of 1:1000 in normal growth medium. After 1 h the cells were trypsinised, washed with PBS and counted in a Bürkner chamber. The cells were fixed in ice cold 70% ethanol. Fixed cells were incubated with 2M HCl containing 0.2mg/ml pepsin for 20 minutes to digest the cell proteins. After washing, the samples were incubated with blocking buffer (1% BSA, 0.5% Tween-20 in PBS). The samples were incubated with Alexa Fluor® 488 labeled BrdU mouse monoclonal antibody (Clone MoBU-1) (Cat. no. B35139 from Invitrogen) at a concentration 1:60 in blocking buffer and incubated at RT for 1 h with gentle mixing. Samples were washed with PBS and incubated with 5µl of 7-AAD Cell Viability Solution (Cat. no. 559925, BD, Franklin Lakes, NJ) in PBS, in the dark over night at 4° C. The cells were analyzed on a CyFlow® Space Partec Flow cytometer.

### Western blot

Protein lysates of three biological triplicates were harvested using M-PER Mammalian Protein Extraction Reagent (Pierce Scientific, Thermo Fisher Scientific Inc.) supplemented with Halt^TM^ Protease inhibitor cocktail (1:100), (Thermo Fisher Scientific Inc. Cat. no. 87785) and 0.5 mM EDTA. Protein concentration was measured on Nanodrop and equal amount of the protein samples were loaded on a NuPAGE ^®^Novex 4-12% Bis-Tris precast gels (Cat. no. NP0321BOX, Life Technologies, Carlsbad, CA). The proteins were transferred to an Immobilon^®^-P Transfer Membrane, PVDF (Cat. no. IPVH00010, EMD Merck Millipore Corporation, Billerica, MA). The membranes were incubated with FOXO1 (C29H4), Rabbit monoclonal antibody at concentration 1:500 (#2880, Cell Signaling Technology Inc, Danvers, MA). GAPDH (GAPDH, mouse monoclonal, MAB374, Merck Millipore, Billerica, MA), was used as loading control. Signals from the HRP coupled antibodies were generated by ECL^TM^ Prime Western Blotting Detection Reagent (RPN2232 from GE Healthcare) and detected using a CCD camera (LAS-3000, Fujifilm, Tokyo, Japan) and ChemiDoc™MP Imaging System (Bio-Rad Laboratories, Hercules, CA). Band intensities were quantified using ImageJ software and normalized to GAPDH.

### Target site blockers

There are two predicted binding sites for miR-96 in the FOXO1 3’ untranslated region (3'UTR) at location 264-271 and 2139-2146 (Targetscan Human, Release 6.2, June 2012). Target site blockers were designed to bind to the predicted binding sites and several bases on both sites of the binding sites (Figure S1). BLO_FOXO1_miR96-1: TTACT+TCAC+GGT+TTGAGTG and BLO_FOXO1_miR96-2: CTTGAAC+CAC+GGT+TTCATGA. The + is in front of “Locked Nucleic acids” (LNA^TM^) in the DNA sequences (Exiqon A/S, Vedbaek, Denmark). The target site blockers were co-transfected with the miR-96 mimic (100nM) in three different concentrations, 300nM, 600nM or 1µM. Protein levels of FOXO1 were quantified using western blot analysis. Growth was measured, using the SRB assay after transfection with miR-96 mimic and 600nM of target site blockers.

### Statistical analysis

Results were analyzed using Graphpad Prism 5 and statistical significance was calculated using unpaired, two-tailed t-test unless noted otherwise and p<0.05 was considered significant. Cuzick’s trend test was used to analyze the trend of miR-96 expression in WHO I, II and III in cohort 1. For the survival analysis a Log-rank (Mantel-Cox) test was used. Spearman’s rank correlation was used to analyze the correlation of miR-96 expression to the PSA levels in patient cohort 1 and to the FOXO1 levels in the external dataset.

## Results

### miR-96 expression correlates with clinical parameters

**Figure 1:**
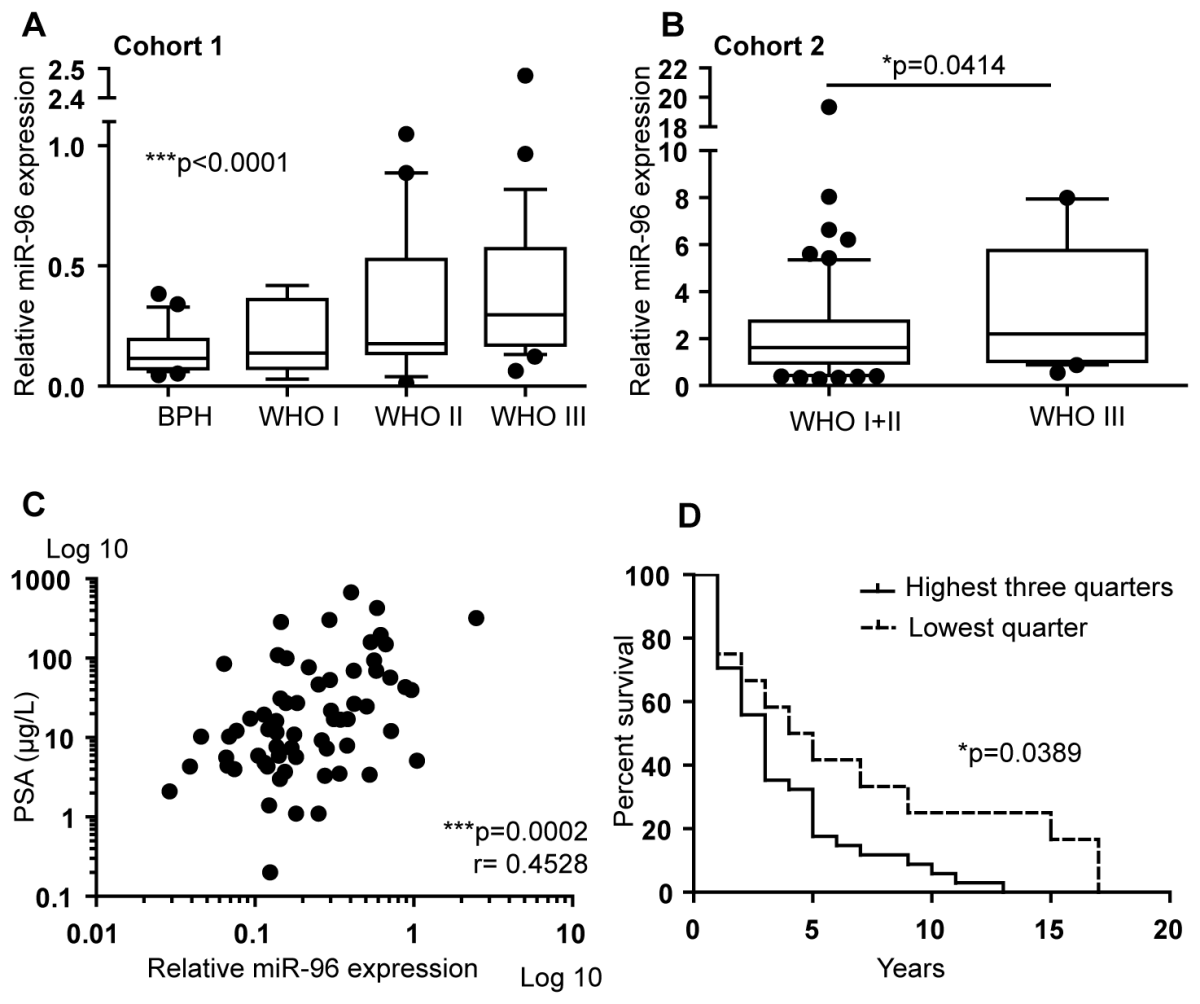
miR-96 expression relative to clinical parameters. **A**. miR-96 expression in the patient samples in cohort 1 is lowest in the BPH (non-PCa) samples and increases significantly with higher WHO grades in the PCa samples (Cuzick’s trend test p<0.0001). When the BPH samples are excluded, the increase in the PCa samples alone is still significant (Cuzick’s trend test, p=0.0498). **B**. In cohort 2, miR-96 expression is significantly higher in PCa patients samples with grading WHO III compared to patient samples with grading WHO I and WHO II combined (t-test p=0.0414). **C**. Increased PSA levels correlate with increased miR-96 expression levels in the patient samples in cohort 1 (p=0.0002, Spearman r=0.4528). **D**. Kaplan-Meier curve showing survival relative to miR-96 expression in cohort 1. The patient group with high miR-96 levels (solid line) has median survival of 3 years and the group with low miR-96 levels (dotted line) has median survival of 4.5 years. Hazard ratio is 2.2. X-axes shows time in years and Y-axes shows percentage survival (Log rank (Mantel-Cox) test p=0.0389).

The levels of miR-96 have previously been found to be significantly higher in PCa tissue than in the non-PCa tissues in cohort 1 [11]. Here, the prognostic properties of miR-96 levels, measured by qRT-PCR on RNA extracted from FFPE prostatic tissues, were investigated. The clinical characteristics of cohort 1 have been thoroughly described previously [31,32] but a shorter version can be seen in table S1. We found miR-96 level to be lowest in BPH (non-PCa) and increase with higher WHO grade, the median miR-96 expression in BPH = 0.1150, WHO I = 0.1368, WHO II = 0.1767, WHO III = 0.2969 (p< 0.0001, Cuzick’s trend test), as seen in Figure 1A. When the PCa samples are compared without including the BPH samples the miR-96 expression increase with WHO grade is still significant (p=0.0498, Cuzick’s trend test). This was also confirmed in a second independent cohort of 93 Swedish men with PCa; the median miR-96 expression in WHO I = 1.620, WHO II = 1.610, WHO III = 2.205. There are only 6 men in the WHO I group, but if WHO I and II are combined, the miR-96 levels in WHO III is significantly higher (p=0.0414) (Figure 1B). Clinical characteristics of cohort 2 are shown in table S2. Increased miR-96 expression correlates with increased PSA levels in patient samples in cohort 1 (Figure 1C). A Kaplan-Meier analysis of patient overall survival in cohort 1 was done based on miR-96 expression levels. Lowest expression quarter compared to high expression in three quarters of the patient samples, significantly divides the PCa patients into high risk (median survival of 3 years) and low risk patients (median survival of 4.5 years) (p=0.0389, log-rank test), with a hazard ratio of 2.2 (95% CI 1.040-4.463), see Figure 1D. Since cohort 2 is a more recent cohort an analyses with survival as endpoint is not possible yet.

### miR-96 expression in tissues and cell lines

**Figure 2:**
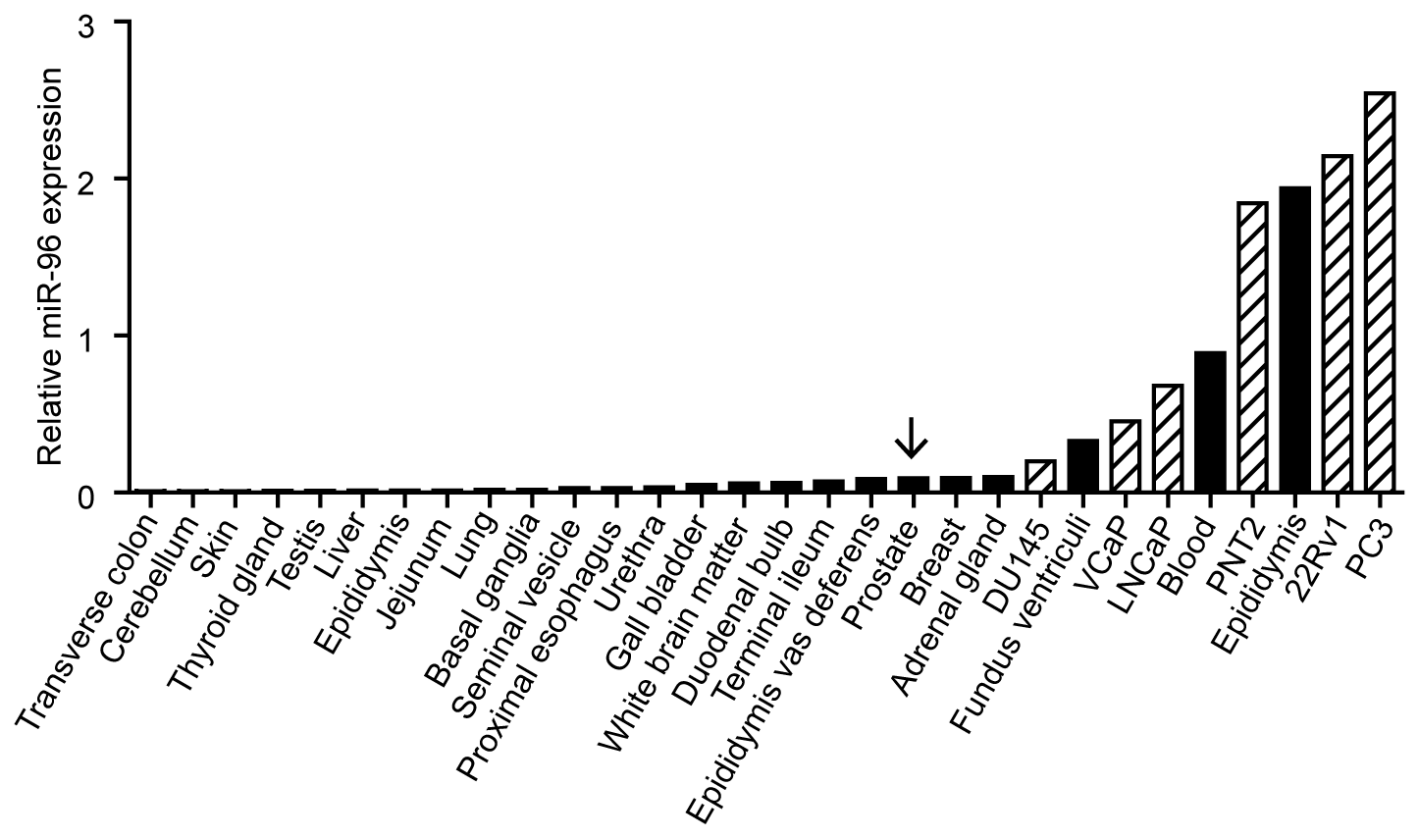
Expression levels of miR-96 in tissue samples and PCa cell lines. miR-96 expression levels in human tissue samples (black bars) and PCa cell lines (striped bars) were measured by qRT-PCR and normalized to the geometric mean of RNU48, RNU66, RNU24 and RNU44. Highest expression was seen in the cell lines and the prostate (arrow) had high expression compared to other tissues.

The miR-96 expression was measured in tissue samples of various origins and in six PCa cell lines (22Rv1, LNCaP, VCaP, DU145, PNT2 and PC3). In the tissue samples, high miR-96 expression was detected in epididymis, blood, adrenal glands and the prostate. The levels in the PCa cell lines were higher than in the normal prostate, with the highest expression in the PC3 and lowest in DU145 cells (Figure 2).

### miR-96 increases cell number and cell growth in PCa cells through cellular proliferation

**Figure 3:**
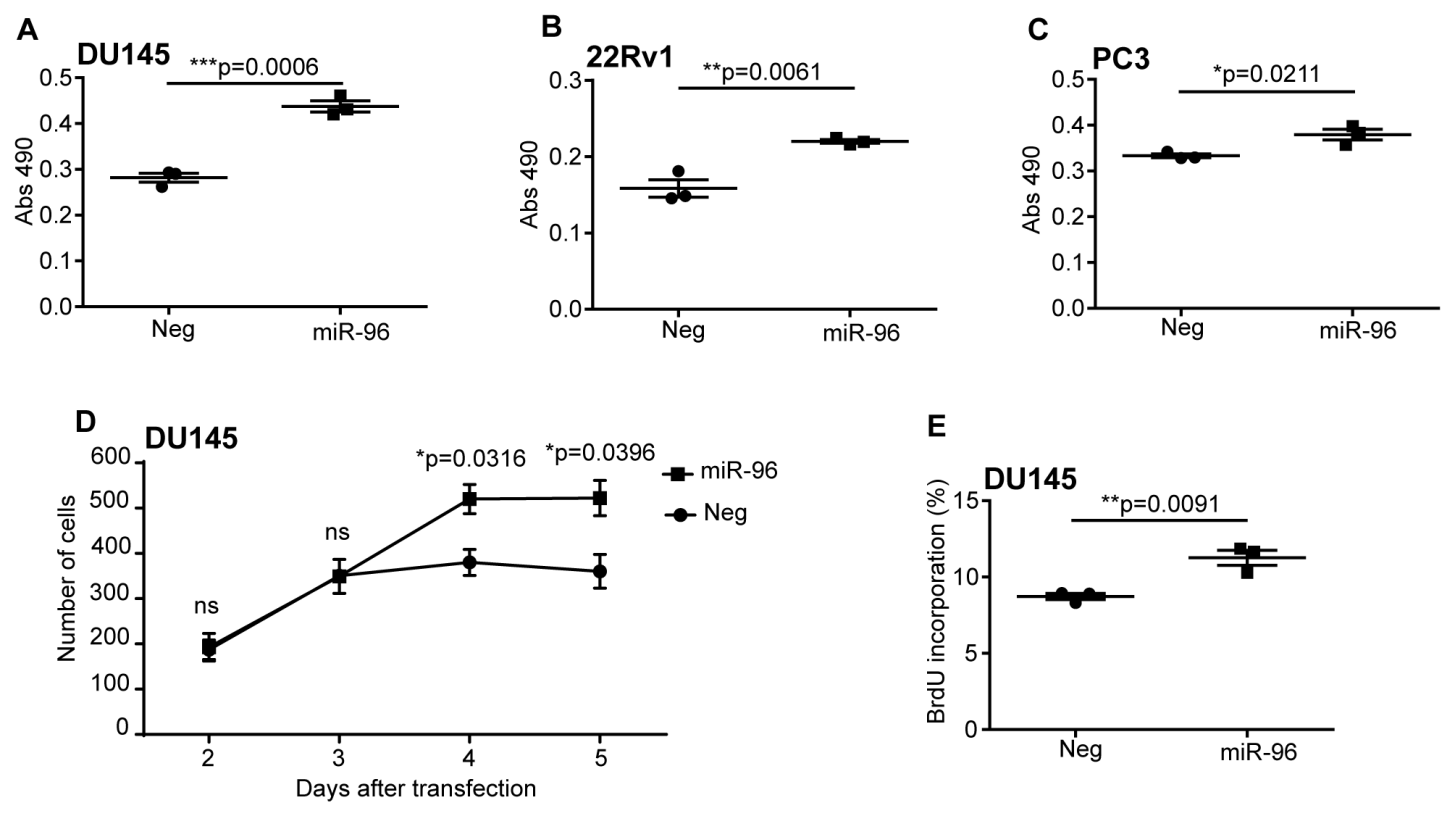
miR-96 increases cell growth and cell number in PCa cells. Ectopic expression of miR-96 increases cell growth in PCa cells. **A**. DU145 cells (p=0.0006). **B**. 22Rv1 cells (p=0.0061). **C**. PC3 cells (p=0.0211). Growth was measured using an SRB assay. **D**. Ectopic expression of miR-96 increases cell number in DU145 cells four (p=0.0316) and five (p=0.0396) days after transfection. **E**. Proliferation is significantly increased in DU145 cells upon overexpression of miR-96(p=0.0091), measured using BrdU incorporation and 7-AAD on a flow cytometer. The mean is represented by a vertical line and error bars show standard error of mean. Results were analyzed using unpaired, two-tailed t-test.

We continued to investigate the biological role of miR-96 in PCa cells *in vitro*. Ectopic expression of miR-96 in different prostate cancer cell lines increased cell growth as measured by a SRB assay; in DU145 cells (p=0.0006), 22Rv1 cells (p=0.0061) and PC3 cells (p=0.0211) (Figure 3A, B and C respectively). It is to be noted, however, that inhibiting miR-96 with miRCury LNA inhibitors in DU145, PC3 or 22Rv1 did not result in change in cell growth as measured by SRB (data not shown). The effect of miR-96 on cell growth corresponded to an effect on cell number of DU145 cells, as measured by cell counting. The ectopic expression of miR-96 significantly increases the cell numbers four (p=0.0316) and five (p=0.0396) days after transfection (Figure 3D). This was shown to be due to an increase in proliferation, as an ectopic expression of miR-96 significantly increased the BrdU incorporation in DU145 compared to the negative control (p=0.0091) (Figure 3E). We did not detect a significant shift in cells in G1, G2 or S-phase between the cells transfected with miR-96 and the negative control.

### miR-96 overexpression decreases the FOXO1 mRNA and protein levels

**Figure 4:**
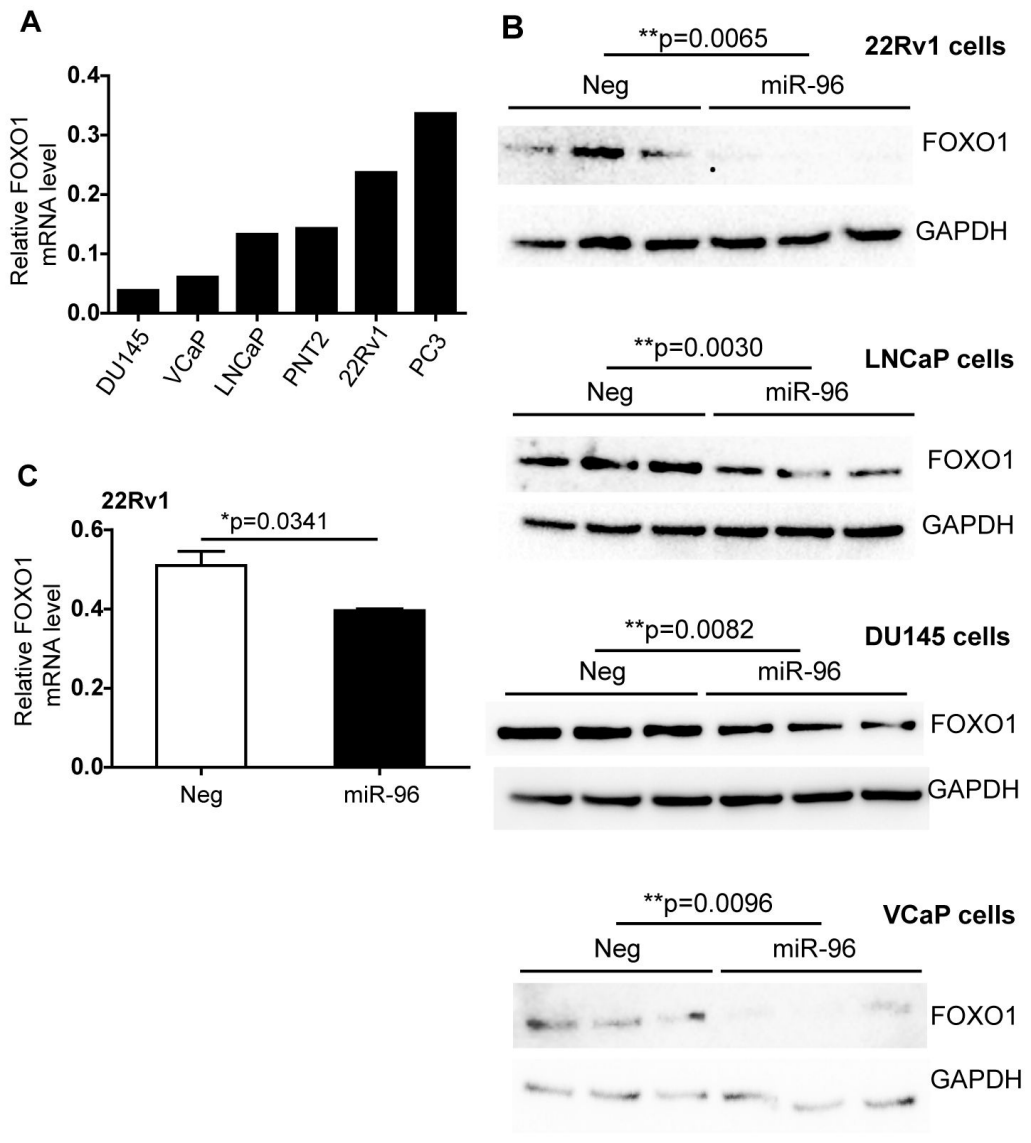
miR-96 significantly decreases FOXO1 mRNA and protein levels in PCa cells. **A**. Endogenous FOXO1 mRNA levels in PCa cell lines. mRNA levels were measured by qRT-PCR and normalized to the mean of GAPDH and PGK1. **B**. FOXO1 protein levels are significantly decreased in 22Rv1 cells (p=0.0065), LNCaP cells (p=0.0030), DU145 cells (p=0.0082) and VCaP cells (p=0.0096) upon miR-96 overexpression. The samples show biological triplicates and the FOXO1 protein levels were normalized to GAPDH protein levels. **C**. FOXO1 mRNA levels are significantly decreased in 22Rv1 cells after overexpression of miR-96(p=0.0341). Measured by qRT-PCR and normalized to the mean of GAPDH and PGK1. Error bars show standard error of mean.

**Figure 5:**
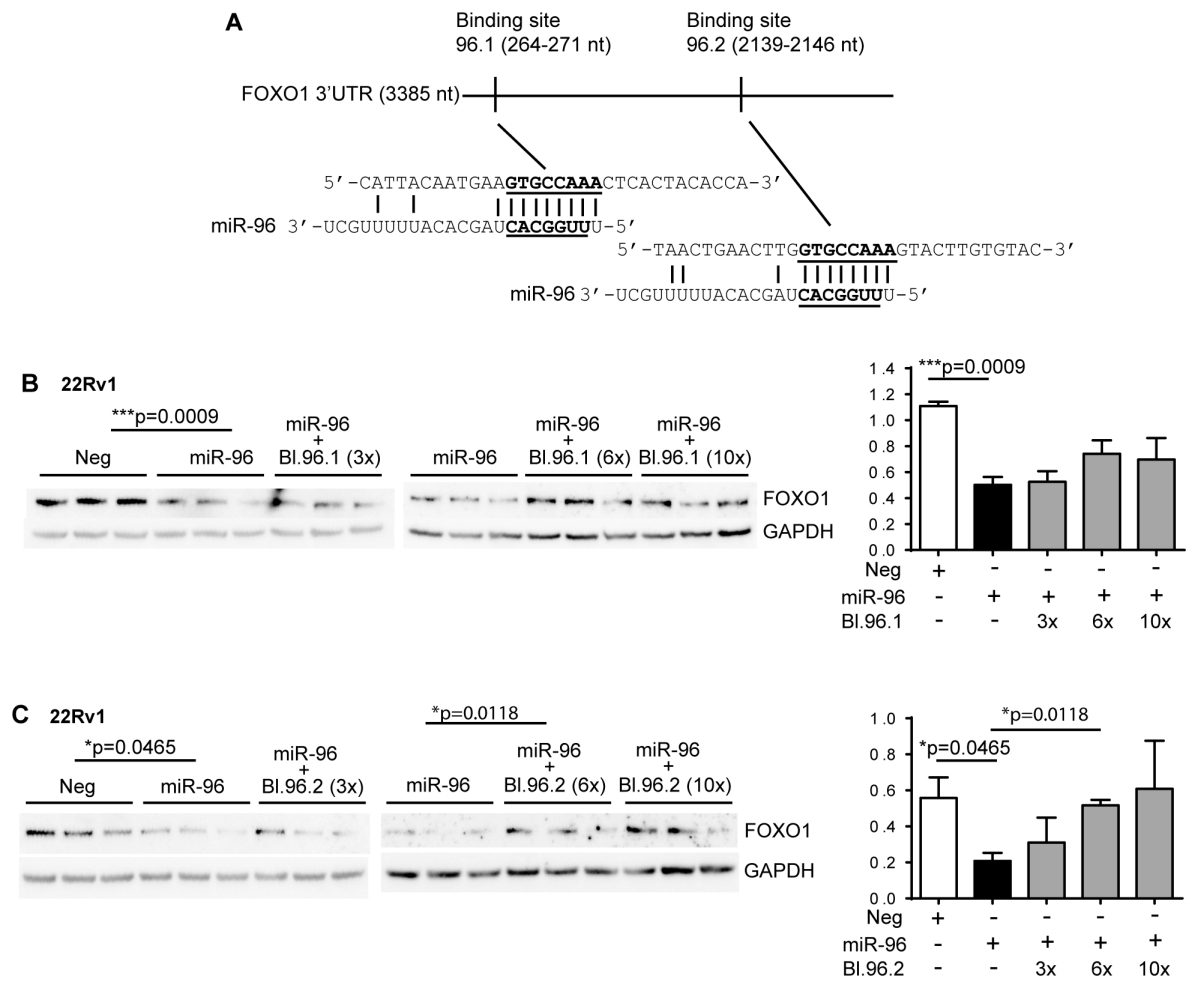
miR-96 binds to the second predicted binding site (96.2) in the 3'UTR of FOXO1 to decrease the protein level. **A**. There are two predicted miR-96 binding sites in the FOXO1 3'UTR sequence. The seed region of the mature miR-96 and the predicted binding sites in the 3'UTR sequence are underlined and bold. Locations of the binding sites are according to Targetscan, (Release 6.2, June 2012). **B**. 22Rv1 cells were co-transfected in triplicates with miR-96 mimic and a target site blocker for binding site 96.1 in three concentrations. FOXO1 protein level did not increase when binding site 96.1 was blocked. **C**. Blocking binding site 96.2 with 6x the concentration of target site blocker compared to the miR-96 mimic resulted in a significant increase of the FOXO1 protein level (p=0.0118). FOXO1 protein levels were normalized to GAPDH. Error bars show standard error of mean.

**Figure 6:**
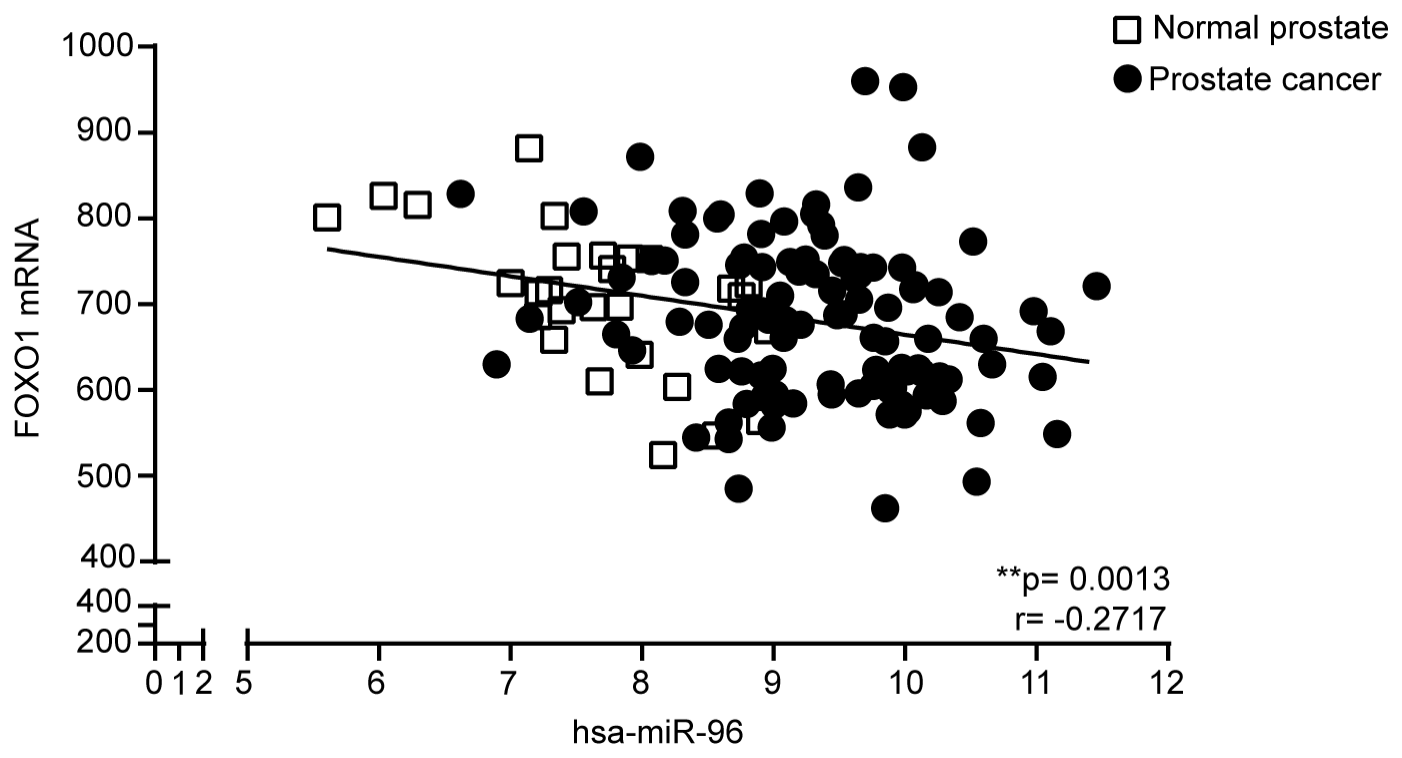
FOXO1 mRNA levels correlate inversely to miR-96 expression levels in PCa and adjacent non-malignant tissue samples. FOXO1 mRNA levels inversely correlate to miR-96 expression levels in an external dataset [34]. The dataset contains 110 PCa tissue samples and 28 non-malignant benign prostate tissue samples (GEO accession number GSE21036) (p=0.0013; Spearman r= -0.2717).

As it has been shown in other cancer types that miR-96 can regulate FOXO1 levels and FOXO1 has been described to inhibit proliferation this would explain the proliferative phenotype of miR-96. We therefore set out to investigate the effect of miR-96 on FOXO1 in PCa cells. First we investigated the endogenous FOXO1 levels in the prostate cell lines. The highest expression of FOXO1 was found in 22Rv1 and PC3 and lowest in DU145 cells (Fig. 4A). We chose 22Rv1, LNCaP and DU145 as model systems. Overexpressing miR-96 significantly reduced the FOXO1 protein levels in 22Rv1 cells (p=0.0065), LNCaP cells (p=0.0030) and DU145 cells (p=0.0082) (Fig. 4B). The FOXO1 levels are also decreased upon miR-96 overexpression in VCaP cells (p=0.0096) although the endogenous levels of FOXO1 are low in this cell type (Fig. 4B). Since the decreasing effect of miR-96 on FOXO1 was most pronounced in 22Rv1 cells, we decided to investigate the FOXO1 transcription level in this cell line. We found that miR-96 overexpression significantly lowered FOXO1 mRNA levels in 22Rv1 cells (p=0.0341) (Fig. 4C). This was however not as pronounced as the effect on protein levels, indicating that miR-96 is acting both by degrading the FOXO1 transcript and blocking the protein translational. FOXO1 contains two *in silico* predicted binding sites for miR-96 (Fig. 5A). To investigate whether miR-96 binds directly to both of these sites, the effect of blocking each of the two predicted binding sites was analyzed using target site blockers specifically designed for each binding site (fig. S1) and co-transfected with miR-96 mimics in 22Rv1 cells. Using anti-FOXO1 antibody and comparing the band intensities to GAPDH, no significant increase in FOXO1 protein level was observed when the predicted binding site 96.1 was blocked, (Figure 5B). However, the protein level increased significantly when the second binding site, 96.2 was blocked, using 6-fold concentration of the blocker compared to the miR-96 mimics (Figure 5C). This indicates that the effect of miR-96 was dependent on access to the second site to be able to decrease the FOXO1 levels. Of note is also that when both targets sites blockers were combined the effect was lost. The regulation of FOXO1 was further corroborated in an external dataset of 110 PCa patients and 28 non-malignant benign prostate tissue samples [34], were miR-96 expression inversely correlates to the expression of FOXO1 in the prostate cancer tissue samples (p=0.0193, Spearman, r=-2228) and in the non-malignant tissue samples (p=0.0029, Spearman, r=-0.5424) separately, as well as when all samples are combined (p=0.0013, Spearman, r=-0.2717) (Figure 6).

### The effect of miR-96 on cell growth is mediated by FOXO1

**Figure 7:**
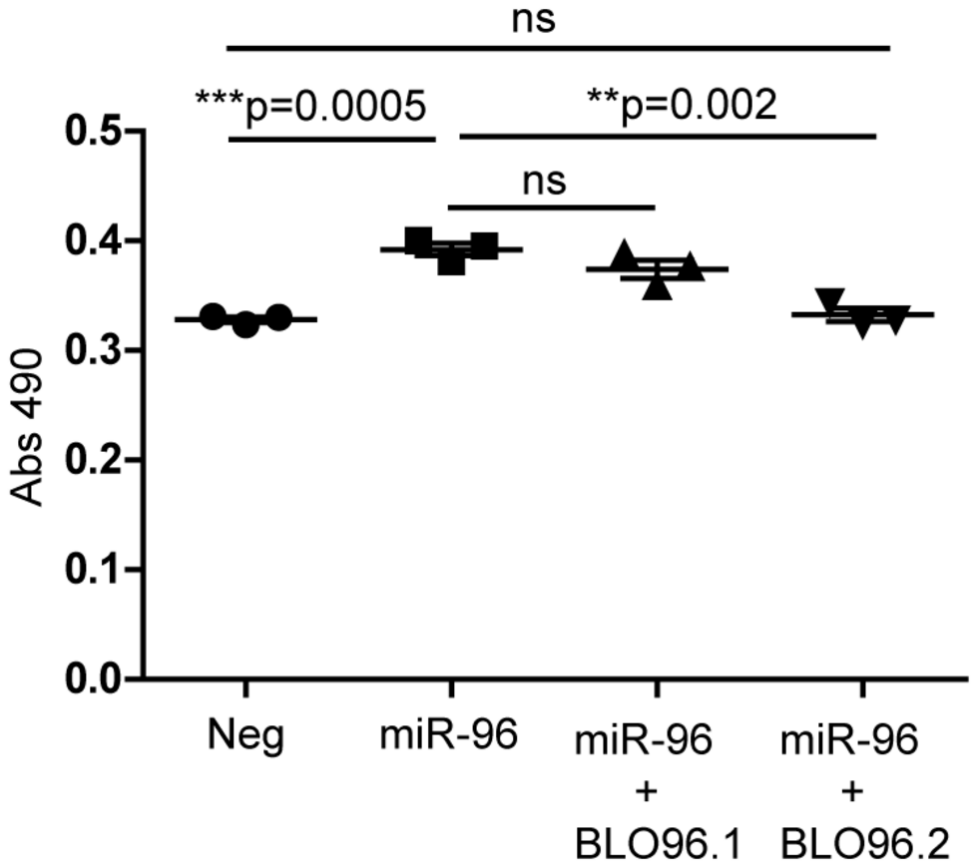
Blocking binding site 96.2 in the FOXO1 3'UTR sequence completely inhibits the cell growth increase by miR-96. miR-96 enhances growth in DU145 cells significantly (p=0.0005). Blocking the second miR-96 binding site (96.2) in the FOXO1 3'UTR sequence with a target site blocker completely eliminates the effect of miR-96 on the cell growth (p=0.002). Blocking the first miR-96 binding site (96.1) does not inhibit the effect of miR-96 on the cell growth. Cell growth was measured using an SRB assay. The mean is represented by a vertical line and error bars show standard error of mean.

We next set out to investigate if FOXO1 is the main target conveying the growth promoting miR-96 phenotype in prostate cells. Interestingly, by blocking the second miR-96 binding site (96.2) in the FOXO1 3’ UTR sequence with the target site blocker, miR-96 is no longer able to enhance the growth of the DU145 cells as measured by an SRB assay (Figure 7). This strengthens the hypothesis that the effect of miR-96 on the cell growth is promoted exclusively through FOXO1. Blocking the first miR-96 binding site did not decrease the growth significantly which further confirms the finding that miR-96 uses the second miR-96 binding site to inhibit FOXO1.

## Discussion

High levels of miR-96 have been detected in prostate cancer [5,7,11] and several studies indicate the importance of miR-96 in prostate cancer progression. In the present study, we show that increased miR-96 expression associates with progression of PCa as the miR-96 expression increases with increased WHO grade in cohort 1. This is not seen in cohort 2 which is a more homogenous cohort of radicals constituting of 63% WHOII. Further, the overall survival is significantly shorter in the patients that have the highest miR-96 levels. This has also been shown for miR-96 expression in lung cancer patient tumor and serum samples. The miR-96 expression level correlates with poor post operative survival [35]. *In vitro*, we find that overexpression of miR-96 in PCa cell lines results in increased growth and proliferation, further confirming the involvement of miR-96 in PCa progression. Few targets of miR-96 have been identified that explain the effect of miR-96 on PCa cells. In endometrial [16], breast [17] and hepatocellular cancer [18], miR-96 has been shown to target the tumor suppressor FOXO1. FOXO1 has been reported to be decreased in PCa [24,25] and inhibition by miR-96 may contribute to this. Here we identify FOXO1 as a target of miR-96 in four different PCa cell lines with both high endogenous FOXO1 levels (22Rv1) as well as low endogenous levels (VCaP), indicating that miR-96 is a potent inhibitor of FOXO1 protein production. Our data indicate that the regulation is occurring at both transcriptional and translational levels, as mRNA and to even greater extent the protein level were affected. That the main regulation is occurring at translational level could be one explanation to the fact that the expression levels of miR-96 and FOXO1 mRNA in the prostate cell lines are positively correlated, with the lowest levels in DU145 and with highest expression for both found in PC3 cells. Hypothetically the trend corresponds to the amount of miR-96 needed in each cell type in order to maintain low FOXO1 protein levels. miR-96 has been shown to decrease the mRNA level of FOXO1 significantly in hepatocellular carcinoma cells and decrease the protein levels slightly [18]. Here, we show in a PCa cell line 22Rv1, that miR-96 binds only to the second of the two predicted binding site (96.2) in the 3’ UTR of FOXO1. In contrast, the miR-96 regulation in breast cancer cells was shown to be dependent on access to both binding sites [17]. miR-96 has been described to possess both oncogenic and tumor suppressive properties depending on the cellular setting, inducing apoptosis in pancreatic cancer cells and promoting proliferation in e.g. breast cancer cell, suggesting that the miR-96 action can be cell specific. The cellular setting might be of relevance for the efficiency of the miR-96 regulation of FOXO1, due to the 3D structure of the mRNA, the presence or absence of other RNA binding proteins *etc*. We show that miR-96 induces cell growth and proliferation through FOXO1. We show that blocking the second binding site (96.2) is sufficient to completely eliminate the growth increase brought on by the miR-96 expression, clearly showing that miR-96 binding to the 96.2 binding site of FOXO1 is sufficient to increases PCa cell growth and proliferation. Blocking both predicted binding sites simultaneously did not show increased effect on neither FOXO1 levels nor growth, indicating that there is no synergistic effect between the two binding sites in PCa cells. It is interesting that FOXO1 can repress the activity of AR in PCa cells [24,29,30], indicating that FOXO1 can be a potential therapeutic target for castration resistant PCa [36]. Loss of FOXO1 would then lead to increased AR activity and subsequently to increased transcription of AR regulated genes such as PSA. We show here that miR-96 levels correlate to the levels of PSA in PCa patients, and we tested whether overexpressing miR-96 could increase AR activity, by measuring the PSA levels in PCa cells. Unfortunately, we were unable to confirm a direct increase of PSA levels in LNCaP cells upon miR-96 overexpression (data not shown). One explanation could be that miR-96 on its own is not enough to induce this effect and that other factors involved in AR regulation might be necessary. It has been suggested that the suppression of AR is mediated through PTEN [37], and the LNCaP cells do not have a functional PTEN. Another explanation could be that upon miR-96 induced repression of FOXO1 leading to increased AR activity, the FOXO1 effect is diminished by the reciprocal inhibition of FOXO1 activity.

To summarize, in this study we find increased expression of miR-96 in PCa and miR-96 shows oncogenic activity by increasing growth and proliferation in PCa cells. miR-96 can decrease the protein levels of FOXO1 through a binding site in the 3’ UTR resulting in increased PCa cell growth and proliferation. The results indicate that targeting miR-96 levels could potentially be beneficial as a novel therapeutic strategy in PCa.

## Supporting Information

Table S1 **Clinical characteristics of cohort 1.** Cohort 1 comprises of tissue samples collected from transurethral resection of the prostate (TURPs), collected 1990-1999 in Malmö, Sweden. The cohort consists of tissue samples from 49 PCa patients and 25 men with BPH (non-PCa).
(TIF)

Table S2 **Clinical characteristics of cohort 2.** Cohort 2 comprises of 93 formalin fixed paraffin embedded (FFPEs) tissues obtained from radical prostatectomies, collected at Malmö Hospital 1999–2002.
(TIF)

Figure S1 **Target site blockers for the predicted binding sites in the FOXO1 3'UTR sequence.** Target site blockers were designed to bind to the two predicted miR-96 binding sites 96.1 and 96.2 in the FOXO1 3’ UTR sequence. Underlined and bold are the predicted binding sites and stars represent the “Locked Nucleic Acids” (LNA^TM^) in the target site blockers.
(TIF)
