# Supplementary figures and images for: Upregulation of miR-96 Enhances Cellular Proliferation of Prostate Cancer Cells through FOXO1
Source: PLoS One. 2013 Aug 12;8(8):e72400. doi: 10.1371/journal.pone.0072400 (PMC3741168; doi:10.1371/journal.pone.0072400)

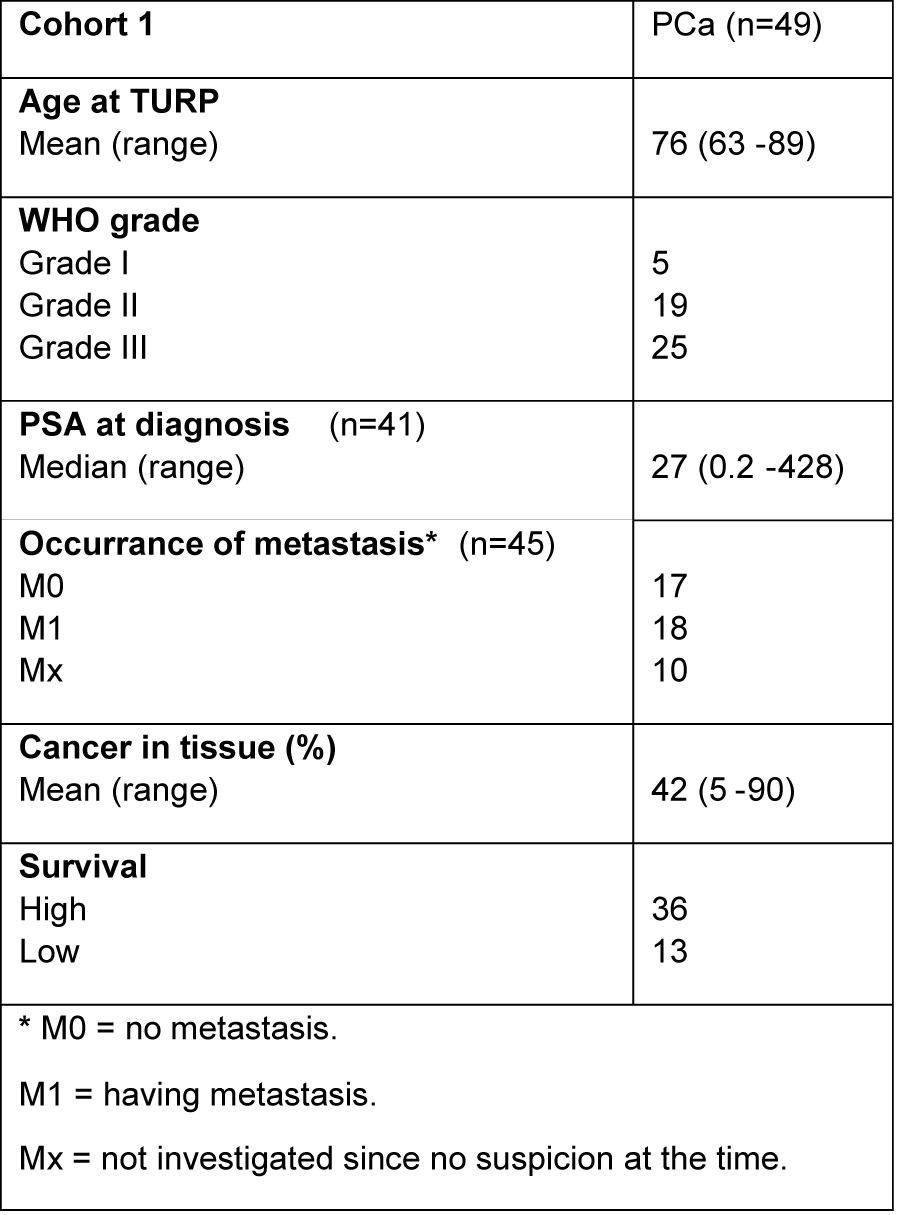

Supplement: Table S1 — Clinical characteristics of cohort 1. Cohort 1 comprises of tissue samples collected from transurethral resection of the prostate (TURPs), collected 1990-1999 in Malmö, Sweden. The cohort consists of tissue samples from 49 PCa patients and 25 men with BPH (non-PCa). (TIF) [file pone.0072400.s001.tif]

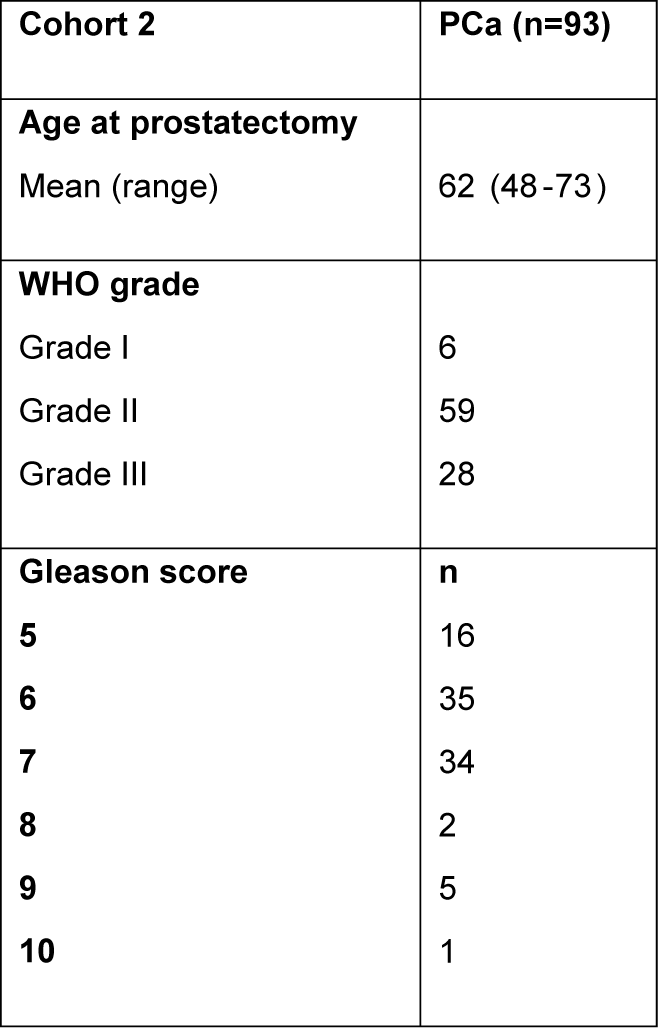

Supplement: Table S2 — Clinical characteristics of cohort 2. Cohort 2 comprises of 93 formalin fixed paraffin embedded (FFPEs) tissues obtained from radical prostatectomies, collected at Malmö Hospital 1999–2002. (TIF) [file pone.0072400.s002.tif]

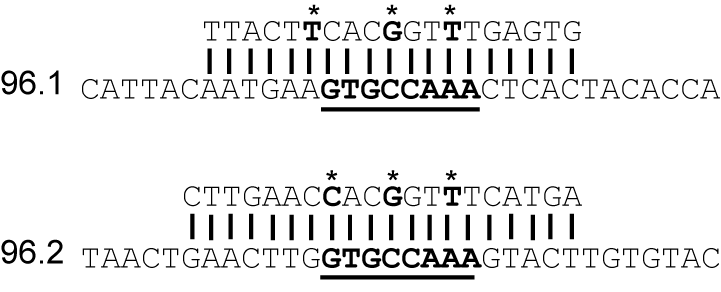

Supplement: Figure S1 — Target site blockers for the predicted binding sites in the FOXO1 3'UTR sequence. Target site blockers were designed to bind to the two predicted miR-96 binding sites 96.1 and 96.2 in the FOXO1 3’ UTR sequence. Underlined and bold are the predicted binding sites and stars represent the “Locked Nucleic Acids” (LNATM) in the target site blockers. (TIF) [file pone.0072400.s003.tif]
